# Impact of achievement and change in achievement of lifestyle recommendations in middle-age on risk of the most common potentially preventable cancers

**DOI:** 10.1016/j.ypmed.2021.106712

**Published:** 2021-12

**Authors:** Juliet A. Usher-Smith, Christel Häggström, Patrik Wennberg, Kristina Lindvall, Jean Strelitz, Stephen J. Sharp, Simon J. Griffin

**Affiliations:** a The Primary Care Unit, Department of Public Health and Primary Care, University of Cambridge School of Clinical Medicine, Box 113 Cambridge Biomedical Campus, Cambridge CB2 0SR, UK; b Department of Public Health and Clinical Medicine, Family Medicine, Umeå University, Umeå, Sweden; c Department of Surgical Sciences, Uppsala University, Uppsala, Sweden; d Department of Epidemiology and Global Health, Umeå University, Umeå, Sweden; e MRC Epidemiology Unit, University of Cambridge, Institute of Metabolic Science, Cambridge CB2 0QQ, UK

**Keywords:** Cancer prevention, Lifestyle, Change, Västerbotten intervention programme

## Abstract

This study aimed to assess the association between achievement, and within-person change in achievement, of lifestyle recommendations in middle-age and incidence of the most common potentially preventable cancers. We used data from 44,572 participants from the Swedish Västerbotten Intervention Programme who had attended at least two health checks 9–11 years apart. We assessed the association between the mean number of healthy lifestyle recommendations achieved (lifestyle score), and change in lifestyle score between the health checks, and risk of one or more of the eight most common potentially preventable cancers using Cox regression. Participants were followed-up for 11.0 (SD 4.9) years. A higher mean lifestyle score was associated with a lower hazard of cancer in men (HR 0.81 (95%CI 0.74–0.90) per unit increase) and women (HR 0.90 (0.84–0.96)). There was no evidence of a linear association between change in lifestyle score and risk (HR 0.93 (0.85–1.03) and HR 1.004 (0.94–1.07) per unit change for men and women respectively). When comparing those with an increase in lifestyle score of ≥2 with those who improved less or declined in achievement the HR was 0.74 (0.54–1.00) and 1.02 (0.84–1.24) for men and women respectively. These findings support the inclusion of lifestyle recommendations in cancer prevention guidelines. They further suggest that interventions to change health behaviours in middle-age may reduce risk of the most common preventable cancers in men, but this association was not observed in women. Strategies to encourage healthy lifestyles earlier in the life course may be more effective.

## Highlights

• Meeting more lifestyle recommendations in middle-age reduces cancer incidence.
• Each additional recommendation met is associated with a 10–19% decrease in rate.
• Change in middle-age to achieve ≥2 more recommendations may reduce cancer rate in men.
• Up to 23% of chosen cancers might be prevented if all men achieved ≥2 more recommendations.
• Change in middle-age is not associated with incident cancer in women.

## 1 Introduction

Many national and international organizations, including the World Cancer Research Fund (World Cancer Research Fund, 2021a), the Swedish Public Health authority (Swedish Public Health Authority, 2021), and the UK Department of Health (Bull et al., 2010; Public Health England, 2016; NHS Choices, 2019), incorporate lifestyle recommendations in guidance to reduce risk of cancer. For many of these recommendations, particularly fruit and vegetable intake, red and processed meat intake and dietary fibre, the evidence comes from prospective cohort studies in which between-individual differences in single lifestyle factors (Aune et al., 2012; Vieira et al., 2015; Liu et al., 2013; Chan et al., 2011; Aune et al., 2011) at one time-point have been associated with risk of specific cancers. The impact of achieving these recommendations on the risk of the most common preventable cancers collectively, and the extent to which change in achievement of these recommendations in middle-age influences future risk of cancer are uncertain.

The evidence for within individual change both for individual and combined cancers is stronger for smoking and alcohol consumption: studies have demonstrated a reduced incidence of cancer in those who have quit or reduced smoking compared with those who continue to smoke (Pirie et al., 2013; Jha et al., 2013; Godtfredsen et al., 2005) and in those who reduce alcohol consumption compared with those who continue (Rehm et al., 2007; Ahmad Kiadaliri et al., 2013). A growing number of studies have also reported changes in cancer incidence following weight change (Harvie et al., 2005; Birks et al., 2012; Rodriguez et al., 2007; Parker and Folsom, 2003; Eliassen et al., 2006; Tee et al., 2013; Rapp et al., 2008) or weight maintenance (Robsahm et al., 2019). However, as highlighted in a report on body fatness and cancer published by the International Agency for Research on Cancer (IARC) in 2016, even for body weight where more than 1000 epidemiological studies have been published, the number and quality of studies reporting data on weight-loss or weight maintenance were judged to be insufficient for formal evaluation (Lauby-Secretan et al., 2016). The evidence for physical activity is also mixed, with studies based on self-reported change in physical activity showing variable associations with individual cancers (Moore et al., 2010; Wolin et al., 2009; Wu et al., 2013) while those based on cardiorespiratory fitness show that a stable or increased cardiorespiratory fitness is associated with lower cancer incidence and mortality (Robsahm et al., 2019; Zhang et al., 2014). There is, therefore, a need for further studies exploring the association between achievement of combined lifestyle recommendations and changes in adherence to lifestyle recommendations at an individual level and future incidence of potentially preventable cancers collectively.

The Swedish Västerbotten Intervention Programme (VIP) (Norberg et al., 2010a; Hallmans et al., 2003) combines population-based strategies with invitations for middle-aged inhabitants to attend individual cardiovascular risk factor screening. Although the primary intention of VIP was not to reduce morbidity and mortality from cancer, repeated lifestyle measures are available for participants and the programme has been associated with decreased smoking prevalence (Norberg et al., 2011), a decrease in the overall trend of increasing obesity (Norberg et al., 2010b) and an increase in physical activity (Ng et al., 2011). It therefore provides a unique opportunity to examine the association between achievement, and change in achievement, of lifestyle recommendations in middle-age and risk of cancer.

We aimed to use data from the VIP cohort to assess the association between achievement of lifestyle recommendations in middle age and within-person change in achievement of lifestyle recommendations in middle-age and risk and population burden of the most common potentially preventable cancers.

## 2 Methods

### 2.1 Population

Within VIP, inhabitants in Västerbotten Country in Sweden are invited to attend a health check at age 40, 50 and 60 years of age. Full details of the programme and the health checks are described elsewhere (Norberg et al., 2010a). For this study eligible participants were individuals within the VIP cohort who had attended at least two health checks between 9 and 11 years apart (hereafter referred to as baseline and 10-year health check) between 1985 and 2008 and who did not have a prior diagnosis of any cancer (excluding basal cell carcinoma) at six months after the date of the 10-year health check. If participants had more than two health checks only the earliest two were used for the analysis.

### 2.2 Outcome

The outcome was a new diagnosis of one or more of the eight most common potentially preventable cancers (lung, bowel, female breast, oesophagus, bladder, kidney, stomach and pancreas) at least six months after the date of the 10 year health check. The eight cancers were identified from published data on the number of cases of each cancer that are potentially preventable in the UK based on estimates of cancer incidence, risk factor prevalence and the published relative risks for risk factors classified by the International Agency for Research on Cancer (IARC) or the World Cancer Research Fund (WRCF) as having ‘sufficient’ (IARC) or ‘convincing’ (WRCF) evidence of a causal association for each cancer (Brown et al., 2018). We excluded melanoma as there is inadequate evidence to suggest that modifiable behaviour in adulthood (such as sun protection habits) can reduce risk (Usher-Smith et al., 2014). Participants with one or more of these cancers were identified through linked data from the Regional Cancer Registry using the ICD-7 codes in Appendix Table A.1. Participants were censored at the date of the first incident cancer. Dates of emigration and of death were retrieved from the population register through the linkage to the Swedish tax agency.

### 2.3 Assessment of lifestyle factors

We considered seven lifestyle factors: tobacco use, physical activity, body mass index (BMI), dietary fibre intake, alcohol intake, red and processed meat consumption, and fruit and vegetable consumption. Table 1 shows which of these risk factors have been associated with which of the eight chosen cancers based on judgements by the WRCF (World Cancer Research Fund, 2021b) and IARC (International Agency for Research on Cancer, n.d.; International Agency for Research on Cancer, 2018).

**Table 1:** Details of which of the chosen lifestyle factors have been associated with which of the eight chosen cancers based on judgements by the WRCF and IARC.

	Lung	Bowel	Breast	Oesophagus	Bladder	Kidney	Stomach	Pancreas
Tobacco use	IARC	IARC		IARC	IARC	IARC	IARC	IARC
Physical activity	WRCF (+)	WRCF (+++)	WRCF (++)	WRCF (+)				
BMI		WRCF (+++) IARC	WRCF (++) IARC	WRCF (+++) IARC		WRCF (+++) IARC	WRCF (++) IARC	WRCF (+++) IARC
Dietary fibre intake		WRCF (+)						
Alcohol intake	WRCF (+)	WRCF (+++) IARC	WRCF (++) IARC	WRCF (+++) IARC		WRCF (++)	WRCF (++)	WRCF (+)
Red/processed meat	WRCF (+)	WRCF (++/+++) IARC					WRCF (+)	WRCF (+)
Fruit and vegetables	WRCF (+)	WRCF (+)	WRCF (+)	WRCF (+)	WRCF (+)		WRCF (+)	

IARC - International Agency for Research on Cancer.

WCRF - World Cancer Research Fund. Level of evidence indicated by +++ convincing, ++ probable, + limited suggestive.

Weight and height were measured. All other factors were self-reported using previously validated measures. Details of how each lifestyle factor was measured and categorised for analysis are given in Appendix B.

We converted each of the seven lifestyle behaviours into dichotomous achievements of recommendations (0 = no, 1 = yes) (Long et al., 2015; Feldman et al., 2017) (Table 2). We then summed them to produce a lifestyle behaviour score ranging from 0 to 7 for both the baseline and 10-year health checks; 0 indicating that no recommendations were achieved, and 7 indicating achievement of all recommendations. For tobacco use, physical activity and BMI we used international recommendations (World Cancer Research Fund, 2021a). For dietary factors, we considered both the Nordic Nutrition Recommendations (Nordic Nutrition Recommendations, 2012) and the Public Health England recommendations (Public Health England, 2016), generating separate scores for each.

**Table 2:** Definitions for achievement of lifestyle recommendations.

Lifestyle factor	Measure	Nordic Recommendations	UK Recommendations
Tobacco use	Smoking status	Never or ex-smoker	
Physical activity	Cambridge physical activity index	At least moderately active	
BMI	kg/m^2^	< 25 kg/m^2^	
Dietary fibre intake	g/day	25–35 g	≥ 30 g
Alcohol intake	g/week	Women <70 g, men <140 g	< 112 g (no more than 14 units of 8 g)
Red and processed meat	g/week	< 500 g	< 500 g (no more than 70 g per day)
Fruit and vegetables	g/day	≥ 500 g	≥ 400 g (5 portions of 80 g)

### 2.4 Statistical analysis

All analyses were performed using Stata (version 15.1) software and stratified by sex.

The association between i) the achievement of each lifestyle factor and mean lifestyle score in the preceding 10 years and ii) within-individual change in achievement of each lifestyle factor and the change in lifestyle score over the preceding 10 years, and risk of one or more of the chosen cancers was assessed using Cox regression. Participants were followed from 6 months after their 10-year health check until the earliest of: date of first diagnosis of one of the chosen cancers; date of emigration; date of death; or date of administrative end of follow-up (31/10/2018). For both analyses, we developed separate models to estimate hazard ratios (HRs) for each of the lifestyle recommendations and for the mean or change in lifestyle score, first for univariate analyses (Model 1), then adjusting for baseline age, sex (male/female), marital status (single/widowed/divorced vs married/partner), education (primary/secondary/university or college) and calendar year (1985–1989/1990–1994/1995–1999/2000–2004/2005–2008) (Model 2) and finally additionally adjusting for the achievement status of all recommendations at the baseline and 10-year health checks (Model 3).

The mean lifestyle score in the preceding 10 years was calculated as the mean of the lifestyle scores at the baseline and 10-year health checks. It was included in the models both as a categorical variable, each value between 0 and 7 representing a separate category with a score of 3 defined as the reference category, and continuously as a score. Achievement of each lifestyle behaviour in the preceding 10 years was categorised into three groups, those achieving the recommendation at: neither baseline nor 10-year health check; at baseline or 10-year health check; and at both baseline and 10-year health check. Those who achieved the recommendation at neither baseline nor 10-year health check were the reference group.

Change in the lifestyle behaviour score over the preceding 10 years was also included both as a categorical variable and a continuous variable with change in unit of the score compared with baseline. For the categorical analyses, two comparisons were performed. In the first, those who met the recommendation at the baseline and 10-year health check (the maintenance group) were compared to those who met the recommendation only at baseline (the no maintenance group). In the second, those who did not meet the recommendation either at the baseline or 10-year health check (the no improvement group) were compared to those who did not meet the recommendation at baseline but did at the 10-year health check (the improvement group). In both cases the reference group was the group with no change (the maintenance group in the first comparison and the no improvement group in the second). The hazard ratios for each comparison were calculated from a single Cox regression model including all four groups using the post-estimation command ‘lincom’ in Stata.

We estimated the population attributable fraction (PAF) for those who achieved a mean lifestyle score of ≥6 in the preceding 10 years compared to those with a lower mean score and for those whose lifestyle score improved in the preceding 10 years ≥1 and ≥ 2 compared to those who improved less or declined in achievement, under the assumption of causality. Both were calculated using the “punafcc” command in Stata (Wolin et al., 2009) based on the most adjusted model.

### 2.5 Sensitivity analysis

In a sensitivity analysis, we investigated the impact on the results of missing data for educational level and marital status at baseline and the seven chosen lifestyle factors at both baseline and follow-up using the Multiple Imputation by Chained Equations (MICE) method (*N* = 20 imputed datasets, Stata command “mi”). The baseline year of health check, age, cancer status and Nelson-Aalen estimate of cumulative hazard were included in the imputation model, with separate imputations for men and women. This method assumes that the data were missing at random.

We also performed a second sensitivity analysis with the Nordic recommendations after removing cases of breast cancer among women to enable comparison between men and women across the same seven cancers (lung, bowel, oesophagus, bladder, kidney, stomach and pancreas).

### 2.6 Ethical approval

Written informed consent was obtained from VIP participants and ethical approval was granted by the Regional Ethical Review Board in Umeå (Nr 2017/08–31 with addendum 2018/390-32 M and 2019/01217).

## 3 Results

**Fig. 1:**
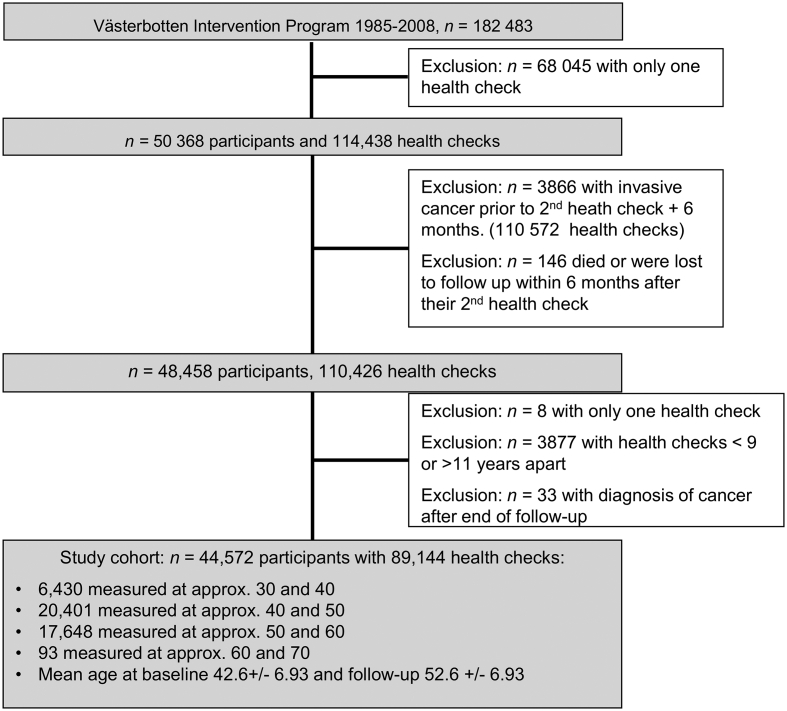
Participant selection.

From 182,483 VIP participants, we included 44,572 in the analysis (Fig. 1).

The mean duration of follow-up after the second health check was 11.0 ± 4.9 years. During that time, there were 1711 (3.8%) incident cases of potentially preventable cancer. The incidence was higher in women (1091, 4.7%), for whom breast cancer and bowel cancer were the dominant cancer types, than in men (620, 2.9%), for whom the dominant cancer types were bowel and bladder (Table 3).

**Table 3:** Incident cases of chosen cancer.

Cancer	Male n (%)	Female n (%)	Total n (%)
Bladder	122 (0.57)	26 (0.11)	148 (0.33)
Bowel	237 (1.10)	209 (0.91)	446 (1.00)
Breast	0 (0)	630 (2.74)	630 (1.41)
Kidney	63 (0.29)	31 (0.13)	94 (0.21)
Lung	84 (0.39)	105 (0.46)	189 (0.42)
Oesophagus	21 (0.10)	6 (0.03)	27 (0.06)
Pancreas	57 (0.26)	59 (0.26)	116 (0.26)
Stomach	36 (0.17)	25 (0.11)	61 (0.14)
Total cases	620 (2.88)	1091 (4.74)	1711 (3.84)

The demographic characteristics of the participants are shown in Table 4 (and stratified by sex in Appendix Table A.2). 38,049 (85.4%) were aged either 40 or 50 at baseline, with the majority of baseline assessments taking place 1990–99. Complete data on all the lifestyle behaviours considered were available at both the baseline and 10 year health checks for 32,767 participants (Table 5). Levels of missing data were highest for dietary factors at baseline (17.8% of participants). The proportion meeting the recommendation for each behaviour at baseline ranged from 80.2% for alcohol consumption to 14.3% for fruit and vegetable consumption. The proportion of participants achieving each recommendation increased between the baseline and 10-year health checks by 7.1% for tobacco use, 6.1% for physical activity, 1.0% for fibre intake, 24.2% for red and processed meat intake, 1.6% for fruit and vegetable intake and 10.9% for alcohol intake. The proportion of participants with a BMI < 25 kg/m^2^ fell by 12.5%. The median lifestyle behaviour score for the Nordic recommendations was 3 (IQR 3–4) at baseline and 4 (IQR 3–4) at 10 years. Similar patterns were seen when considering the UK recommendations (Appendix Table A.3).

**Table 4:** Demographic characteristics of study population at baseline.

	All study participants		No incident cancer		One or more of the chosen cancers	
	n	%	n	%	n	%
Total	44,572	100.0	42,861	100.0	1711	100.0
Age at baseline, years						
30	6430	14.4	6316	14.7	114	6.7
40	20,401	45.8	19,885	46.4	516	30.2
50	17,648	39.6	16,570	38.7	1078	63.0
60	93	0.2	90	0.2	3	0.2
Sex						
Men	21,538	48.3	20,918	48.8	620	36.2
Women	23,034	51.7	21,943	51.2	1091	63.8
Year at baseline						
1985–1989	1974	4.4	1844	4.3	130	7.6
1990–1994	16,350	36.7	15,440	36.0	910	53.2
1995–1999	15,662	35.1	15,122	35.3	540	31.6
2000–2004	6600	14.8	6487	15.1	113	6.6
2005–2008	3986	8.9	3968	9.3	18	1.1
Education at baseline						
Primary	7851	17.6	7404	17.3	447	26.1
Any secondary	24,475	54.9	23,625	55.1	850	49.7
University/College	11,462	25.7	11,098	25.9	364	21.3
Missing	784	1.8	734	1.7	50	2.9
Marital status at baseline						
Single/Widowed/Divorced	7516	16.9	7248	16.9	268	15.7
Married/Partner	36,669	82.3	35,244	82.2	1425	83.3
Missing	387	0.9	369	0.9	18	1.1

**Table 5:** Achievement of Nordic recommendations and lifestyle behaviour score at baseline and 10-year health check.

	All participants (*n* = 44,572)				No incident cancer (*n* = 42,861)				One or more of the chosen cancers (*n* = 1711)			
	Baseline		10-year health check		Baseline		10-year health check		Baseline		10-year health check	
	n	%	n	%	n	%	n	%	n	%	n	%
Tobacco use												
Recommendation met (non-users/past users)	33,814	75.9	36,986	83.0	32,681	76.3	35,695	83.3	1133	66.2	1291	75.5
Missing	784	1.8	645	1.5	760	1.8	616	1.4	24	1.4	29	1.7
BMI, kg/m^2^ (mean, SD)	25.2	3.8	26.5	4.3	25.2	3.9	26.5	4.3	25.2	3.7	26.6	4.3
Recommendation met (<25 kg/m^2^)	23,277	52.2	17,672	39.7	22,415	52.3	16,996	39.7	862	50.4	676	39.5
Missing	679	1.5	41	0.1	645	1.5	36	0.1	34	2.0	6	0.4
Physical activity												
Inactive	7107	15.9	7612	17.1	6839	16.0	7261	16.9	268	15.7	351	20.5
Moderately inactive	13,622	30.6	12,790	28.7	13,047	30.4	12,249	28.6	575	33.6	541	31.6
Moderately active	12,068	27.1	12,538	28.1	11,637	27.2	12,087	28.2	431	25.2	451	26.4
Active	9123	20.5	11,359	25.5	8846	20.6	11,006	25.7	277	16.2	353	20.6
Recommendation met (active/moderately active)	21,191	47.5	23,897	53.6	20,483	47.8	23,093	53.9	708	41.4	804	47.0
Missing	2652	6.0	273	0.6	2492	5.8	258	0.6	160	9.4	15	0.9
Dietary fibre intake, g/day (mean, SD)	19.0	7.4	18.4	7.5	19.0	7.4	18.4	7.6	18.5	6.8	18.4	7.3
Nordic recommendation met (≥25 g/day)	7022	15.8	7502	16.8	6812	15.9	7230	16.9	210	12.3	272	15.9
Missing	7938	17.8	2493	5.6	7517	17.5	2402	5.6	421	24.6	91	5.3
Red and processed meat, g/week (mean, SD)	885.6	611.6	582.9	357.1	884.9	612.6	585.5	358.2	906.3	582.8	519.9	322.2
Recommendation met (<500 g/week)	9588	22.1	20,635	46.3	9573	22.3	19,671	45.9	282	16.5	964	56.3
Missing	7938	17.8	2493	5.6	7517	17.5	2402	5.6	421	24.6	91	5.3
Fruit and vegetable intake, g/day (mean, SD)	315.0	241.2	306.1	233.6	314.4	241.1	305.7	233.7	333.2	243.8	316.3	233.4
Nordic recommendation met (≥500 g/day)	6368	14.3	7065	15.9	6125	14.3	6760	15.8	243	14.2	305	17.8
Missing	7938	17.8	2493	5.6	7517	17.5	2402	5.6	421	24.6	91	5.3
Alcohol intake, g ethanol/week (mean, SD)	29.4	32.1	31.4	34.8	29.5	32.2	31.5	34.9	27.2	30.2	28.8	31.1
Nordic recommendation met (<70 g/week for women; <140 g/week for men)	35,755	80.2	40,621	91.1	34,501	80.5	39,061	91.1	1254	73.3	1560	91.2
Missing	7938	17.8	2493	5.6	7517	17.5	2402	5.6	421	24.6	91	5.3
Nordic lifestyle behaviour score (median, IQR)	3	3–4	4	3–4	3	3–4	4	3–4	3	2–4	4	3–4
≤1	1351	3.0	1018	2.3	1258	2.94	978	2.28	93	5.44	40	2.34
2	6607	14.8	6429	14.4	6342	14.8	6174	14.4	265	15.49	255	14.9
3	11,644	26.1	12,942	29.0	11,210	26.15	12,451	29.05	434	25.37	491	28.7
4	9974	22.4	12,176	27.3	9668	22.56	11,688	27.27	306	17.88	488	28.52
5	4746	10.7	6380	14.3	4626	10.79	6152	14.35	120	7.01	228	13.33
≥6	1679	3.8	2465	5.5	1626	3.79	2382	5.56	53	3.1	83	4.85
Missing	8571	19.2	3162	7.1	8131	18.97	3036	7.08	440	25.72	126	7.36

BMI: Body mass index, IQR: Inter-quartile range, SD: Standard deviation.

**Fig. 2:**
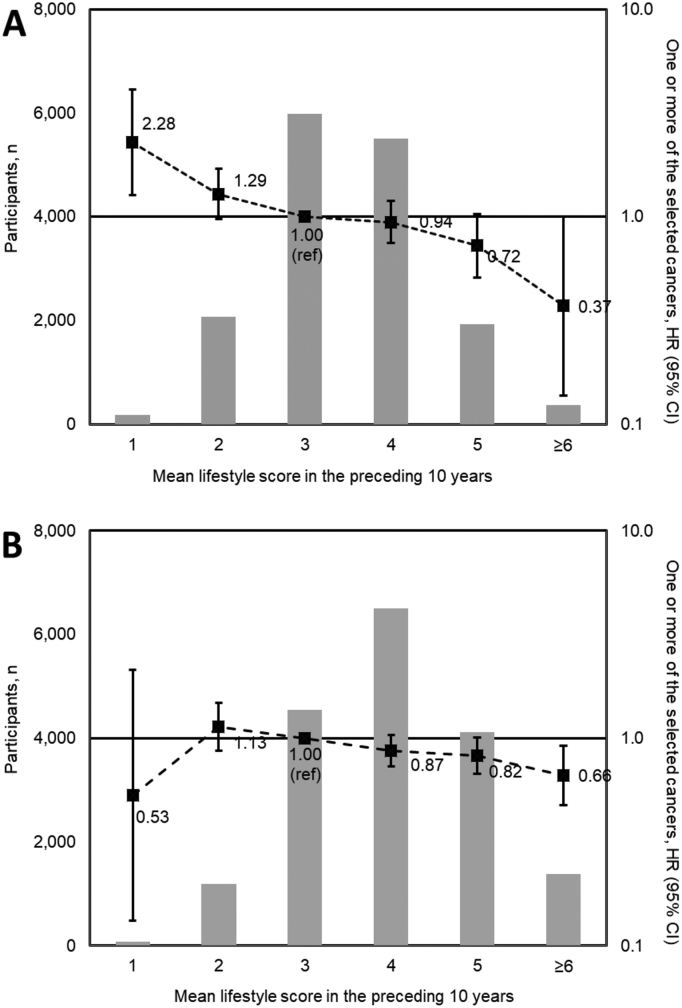
Distribution of mean Nordic lifestyle score in the preceding 10 years (bars, left axis) and association between mean Nordic lifestyle score and cancer incidence (forest plot, right axis in men (A) and women (B). HRs adjusted for age group at baseline, baseline year, education level and marital status. CI confidence interval, HR hazard ratio.

The distribution of mean lifestyle score in the 10 years preceding follow-up and the association with incident cancer is shown in Fig. 2 (data in Appendix Tables A.4a and A.4b). After adjusting for marital status, education, calendar year and age at baseline, a higher mean lifestyle score was associated with a lower hazard of cancer in both men and women. There was a suggestion that the association was stronger for men (HR 0.81 (0.74–0.90) per unit increase in the score) than for women (HR 0.90 (0.84–0.96)). For those with a mean lifestyle score of ≥6 vs those with a mean lifestyle score < 6, the HR for men was 0.41 (0.15–1.10) and the HR for women was 0.75 (0.55–1.02). When excluding breast cancer cases among women, the association in women was comparable with that in men (HR 0.83 (0.75–0.93) and the HR for those with a mean lifestyle score of ≥6 vs those with a mean lifestyle score < 6 was 0.45 (0.24–0.85) (Appendix Tables A.5 and A.6 and Appendix Fig. C.3).

After adjustment for marital status, education, calendar year, age at baseline and achievement status of all other recommendations at baseline and 10-year health check, only smoking status was associated with hazard of cancer (Appendix Tables A.4a and A.4b). Compared with those who were current smokers at both baseline and the 10-year health check, those who were non-smokers at both time points were less likely to develop cancer (HR 0.51 (0.40–0.66) in men and HR 0.78 (0.65–0.95) in women). Findings were similar when considering the UK recommendations (Appendix Fig. C.1 and Appendix Tables A.7a and A.7b)) and in women after excluding breast cancer (Appendix Tables A.5 and A.6 and Appendix Fig. C.3).

**Fig. 3:**
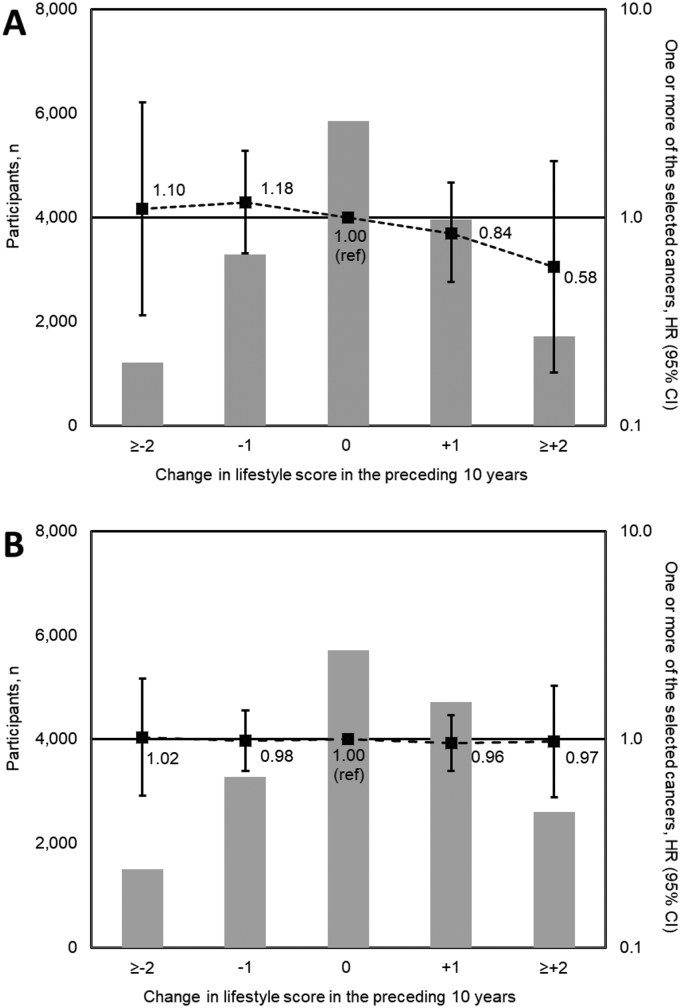
Distribution of change in Nordic lifestyle score in the preceding 10 years (baseline to 10-year health check) (bars, left axis) and association between change in Nordic lifestyle score and cancer incidence (forest plot, right axis in men (A) and women (B). HRs adjusted for age group at baseline, baseline year, education level and marital status. CI confidence interval, HR hazard ratio.

Of the 16,034 men with complete data, 5686 (35.5%) increased their lifestyle score between the baseline and 10-year health checks and 4504 (28.1%) decreased their score. There was no evidence of a linear association (HR 0.93 (0.85–1.03) per unit change of the score) (Fig. 3). However, when comparing men with an increase in lifestyle score of ≥2 with those with an increase <2, the HR was 0.74 (0.54–1.00) (Appendix Table A.8).

Of the 17,805 women with complete data, 7322 (41.1%) increased their lifestyle score between the baseline and 10-year health checks and 4771 (26.8%) decreased their score. There was no association between change in lifestyle score and hazard of cancer (HR 1.004 (0.94–1.07) per unit change of the score). When comparing women with an increase in lifestyle score of ≥2 with those with an increase <2, the HR was 1.02 (0.84–1.24) (Appendix Table A.8).

After adjustment for marital status, education, calendar year, age at baseline and achievement status of all other recommendations at baseline and 10-year health check, only a change in smoking status was associated with hazard of cancer (Appendix Tables A.9a and A.9b). Men who stopped smoking between the baseline and 10-year health checks were less likely to develop cancer than those who continued to smoke (HR 0.64 (0.45–0.92)) and women who started smoking between the baseline and 10-year health checks were more likely to develop cancer than those who remained non-smokers (HR 1.55 (1.03–2.34)). Findings were similar when considering the UK recommendations (Fig. C.2 and Appendix Tables A.8, A.10a and A.10b)) and in women after excluding breast cancer (Appendix Tables A.6 and A.11 and Appendix Fig. C.4).

## 4 Discussion

In this cohort of over 40,000 participants from a community-based cardiovascular disease prevention programme, we have shown that achieving more lifestyle recommendations on average over a 10-year period in middle-age is associated with a lower hazard of the most common preventable cancers in both men and women. There was a suggestion that the association was stronger for men than for women, with achievement of each additional lifestyle recommendation associated with a 19% (10–26%) decrease in hazard in men and a 10% (4–16%) decrease in women.

There was no evidence of a linear association between change in achievement of recommendations and hazard of cancer in either men or women. However, if all men had increased the number of Nordic recommendations they met by two or more we estimated that up to 23% of incident cases of these cancers, and 7% of all cancers, might have been prevented. This association was not seen in women and of the seven individual lifestyle recommendations considered, only cigarette smoking was associated with developing one of the chosen cancers.

The stronger associations in men than women may reflect the different relative distribution of the chosen cancers and the different contributions of lifestyle factors across the cancers. In women, breast cancer accounted for 57.7% (*n* = 630/1091) of incident cases, with bowel cancer (19.2%) and lung cancer (9.6%) the next most common. In men, bowel cancer was the most common (38.2%), followed by bladder cancer (19.7%) and lung cancer (13.5%). While breast cancer has been associated with BMI, alcohol consumption, physical activity and fruit and vegetable consumption, smoking, dietary fibre intake and red/processed meat consumption are also additional established risk factors for bowel cancer, making it the only one of the chosen cancers to be associated with all the included lifestyle factors (Brown et al., 2018). Smoking is also a strong risk factor for lung cancer and bladder cancer, with relative risks from cohort studies assessing between individual differences two to four-fold greater than any of the other lifestyle risk factors for breast cancer (Brown et al., 2018). It is, therefore, not surprising that the overall association between lifestyle factors and hazard of the most common preventable cancers in each sex was found to be different between men and women. For the association between the mean lifestyle score in the previous 10 years and the hazard of cancer, the hazards for men and women were comparable when breast cancer was excluded. This is consistent with previous studies on individual cancers where the relative risks for lifestyle factors are similar between men and women. The difference between men and women for the association between the change in lifestyle score in the previous 10 years and the hazard of cancer persisted after exclusion of breast cancer among women. This may be because of the smaller number of cancer cases in women after exclusion of breast cancer or may reflect a true biological difference between men and women.

The different contributions of lifestyle factors to the chosen cancers likely also explains why only cigarette smoking was associated with developing one or more of the chosen cancers: cigarette smoking is associated with all the chosen cancers except breast cancer and has the highest relative risks for most. This reinforces the importance of smoking as a risk factor for cancer and the benefits of smoking cessation on future hazard of the most common potentially preventable cancers collectively (Pirie et al., 2013; Jha et al., 2013).

The difference in both sexes between the association observed for mean lifestyle achievement and cancer incidence, and the absence of a linear association with change in achievement of lifestyle recommendations, suggests that the reductions in cancer risk over 11 years achievable through individuals changing lifestyle in middle-age are small compared with those associated with between-individual differences. This is consistent with other studies. For example, a large cohort study among 328,781 participants across Europe found that a higher BMI at recruitment (mean age 50 years) was associated with an increase in colon cancer incidence in men (HR 1.04; 95% CI 1.02–1.07) but subsequent weight gain or loss was not related to colon or rectal cancer risk in men or women (Bisschop et al., 2014). Weight gain of 2.0 kg or more since age 18 years has also been estimated to explain 15% of breast cancer cases, with only 4.4% of cases of breast cancer attributable to the same weight gain since menopause (Eliassen et al., 2006). As has previously been suggested, this lack of effect of change in middle-age may be because gaining weight later in life is less detrimental than gaining weight earlier in life (Bisschop et al., 2014). Similar explanations may be the case for the other lifestyle risk factors included in this study.

We considered a number of limitations when interpreting the findings. In particular, there were few incident cancers, particularly among those under 50 years of age. Additionally, less than 20% of participants changed their lifestyle score by two or more between baseline and 10-year health checks and the median lifestyle behaviour score only increased from 3 (IQR 3–4) at baseline to 4 (IQR 3–4) at the 10-year health check. Although small, these changes are comparable with other population-based interventions (Blomstedt et al., 2015; Record et al., 2015), and therefore are likely to reflect the magnitude of changes realistically achievable among middle-age individuals. While we used validated measures, imprecise self-report of lifestyle behaviours may have led to regression dilution bias and introduced recall and social desirability bias. We also considered only seven lifestyle factors and assessed the number of lifestyle recommendations achieved on the basis of the dichotomized value of each lifestyle factor, treating all as equally important and potentially missing small changes in lifestyle insufficient to move between categories. Finally, we cannot exclude residual confounding.

## 5 Conclusion

Our data confirm the association between achievement of lifestyle recommendations and cancer in middle-age (Brown et al., 2018; Li et al., 2018; Li et al., 2020) and support the inclusion of lifestyle recommendations in national and international cancer prevention guidelines. They further suggest that the development and implementation of individual and population-based approaches to change health behaviours in middle-age may reduce risk of the most common preventable cancers in men, but this association was not observed in women. Strategies to encourage the adoption and maintenance of healthy lifestyles earlier in the life course may be more effective.

## Availability of data and materials

All relevant aggregated data are presented in this article. Requests for the individual-level data can be made to the Department of Biobank Research, Umeå University (http://www.biobank.umu.se/biobank/nshds/), and will be subject to ethical review and assessment by a panel of scientists. Individual-level data cannot be made publically available due to legal restrictions imposed by the Swedish Authority for Privacy Protection.

## Funding

JUS was supported by a 10.13039/501100000289Cancer Research UK Cancer Prevention Fellowship (C55650/A21464).

SJS and SJG are supported by grants from the 10.13039/501100000265Medical Research Council (MC_UU_00006/6). The University of Cambridge has received salary support in respect of SG from the NHS in the East of England through the Clinical Academic Reserve. All researchers were independent of the funding bodies and the funders had no role in data collection, analysis and interpretation of data; in the writing of the report; or decision to submit the article for publication.

## Declaration of Competing Interest

The authors declare that there are no competing interests.

## Appendix Supplementary data

Appendix A Appendix Table A.1 ICD7 codes to identify cancers of interest. Appendix Table A.2. Demographics of study population at baseline stratified by sex. Appendix Table A.3. Achievement of UK recommendations and lifestyle behaviour score at baseline and 10 year health check. Appendix Table A.4a. Association between achievement of Nordic lifestyle recommendations in the preceding 10 years and cancer risk in men. Appendix Table A.4b. Association between achievement of Nordic lifestyle recommendations in the preceding 10 years and cancer risk in women. Appendix Table A.5. Association between achievement of Nordic lifestyle recommendations in the preceding 10 years and cancer risk in women, excluding breast cancer. Appendix Table A.6. Linear associations and population attributable fractions for women, excluding breast cancer. Appendix Table A.7a. Association between achievement of UK lifestyle recommendations in the preceding 10 years and cancer risk in men. Appendix Table A.7b. Association between achievement of UK lifestyle recommendations in the preceding 10 years and cancer risk in women. Appendix Table A.8. Linear associations and population attributable fractions. Appendix Table A.9a. Association between cancer risk and change in Nordic lifestyle recommendation achievement between baseline and 10-year health check in men. Appendix Table A.9b. Association between cancer risk and change in Nordic lifestyle recommendation achievement between baseline and 10-year health check in women. Appendix Table A.10a. Association between cancer risk and change in UK lifestyle recommendation achievement between baseline and 10-year follow up in men. Appendix Table A.10b. Association between cancer risk and change in UK lifestyle recommendation achievement between baseline and 10-year follow up in women. Appendix Table A.11. Association between cancer risk and change in UK lifestyle recommendation achievement between baseline and 10-year follow up in women, excluding breast cancer. Appendix A

Appendix B Appendix B. Assessment of lifestyle factors Appendix B

Appendix C Appendix Figure C.1a Distribution of mean UK lifestyle score in the preceding 10 years and association between mean UK lifestyle score and cancer incidence in men. Appendix Figure C.1b Distribution of mean UK lifestyle score in the preceding 10 years and association between mean UK lifestyle score and cancer incidence in women. Appendix Figure C.2a Distribution of change in UK lifestyle score in the preceding 10 years (baseline to 10-year health check) and association between change in UK lifestyle score and cancer incidence in men. Appendix Figure C.2b Distribution of change in UK lifestyle score in the preceding 10 years (baseline to 10-year health check) and association between change in UK lifestyle score and cancer incidence in women. Appendix Figure C.3. Distribution of mean Nordic lifestyle score in the preceding 10 years (bars, left axis) and association between mean Nordic lifestyle score and cancer incidence (forest plot, right axis) in women, excluding breast cancer.. Appendix Figure C.4. Distribution of change in Nordic lifestyle score in the preceding 10 years (baseline to 10-year health check) (bars, left axis) and association between change in Nordic lifestyle score and cancer incidence (forest plot, right axis) in women, excluding breast cancer.. Image 1
