# Supplementary material for: Impact of achievement and change in achievement of lifestyle recommendations in middle-age on risk of the most common potentially preventable cancers
Source: Prev Med. 2021 Dec;153:106712. doi: 10.1016/j.ypmed.2021.106712 (PMC8633845; doi:10.1016/j.ypmed.2021.106712)
Supplement: Appendix B — Assessment of lifestyle factors [file mmc2.pdf]

Appendix B. Assessment of lifestyle factors

Tobacco use

Tobacco use was assessed by self-reported smoking in the VIP questionnaire and classified as daily smokers, ex- smokers (former daily smokers) or never smokers (including occasional smokers and former occasional smokers).

Physical activity

Physical activity was measured using the Cambridge Physical Activity Index, a validated index based on two questions related to physical activity at work and in leisure time[1,2]. Participants were categorised into four groups ranging from inactive to active as in Appendix Table B.1. Where a value was missing in one of the two included variables, the missing value was replaced by the lowest level of activity for that variable. This approach was chosen as the majority of those with missing data are participants who do not work and therefore do not report occupational physical activity.

Category	Description
Inactive	Sedentary job and no recreational activity
Moderately inactive	Sedentary job with <0.5 hour recreational activity per day or Standing job with no recreational activity
Moderately active	Sedentary job with 0.5 to 1 hour recreational activity per day or Standing job with 0.5 hour recreational activity per day or Physical job with no recreational activity
Active	Sedentary job with >1 hour recreational activity per day or Standing job with >0.5 hour recreational activity per day or Physical job with at least some recreational activity or Heavy Manual Job

Appendix Table B.1. Categorisation of physical activity according to the Cambridge Physical Activity Index

Body mass index (BMI)

BMI was calculated from height and weight measurements in light clothing at the health assessments. Values were set to missing for those with values <15 and >70.

Dietary fibre, alcohol intake, red and processed meat consumption, and fruit and vegetable consumption

Dietary fibre, alcohol intake, red and processed meat consumption, and fruit and vegetable consumption were assessed using a modified version of the validated Northern Sweden Food Group

Frequency Questionnaire (FFQ) with 64–84 items[3]. Fruit and vegetables included berries, apple, pear, peach, orange, mandarin, grapefruit, banana, white cabbage, root vegetables, carrot, tomato, cucumber, lettuce, cabbage, spinach, broccoli and mixed frozen vegetables. Red and processed meat included minced meat, meat stew, steak, bacon, sausage, hamburger, sausage on bread and meat on bread. Intake frequency had nine fixed alternatives (never, occasionally, 1–3 times/month, 1 time/week, 2–3 times/week, 4–6times/ week, 1 time/day, 2–3 times/day, ≥4 times/day). Reported frequencies of consumption were converted to number of intakes per day and multiplied by a portion size to derive grams (g)/day or g/week. As in previous studies[4,5], for these dietary variables and alcohol, we excluded participants from the complete case analyses who had food intake levels (calculated by dividing reported total caloric intake with estimated basal metabolic rate) below the 1st percentile or above the 99th percentile calculated separately by sex, missing body weight so that food intake levels could not be calculated, and where >10% of food frequencies were missing and/or portion size indications not complete. These values were set to missing and subsequently imputed in the imputed analyses.

References

[1] Wareham NJ, Jakes RW, Rennie KL, Schuit J, Mitchell J, Hennings S, et al. Validity and repeatability of a simple index derived from the short physical activity questionnaire used in the European Prospective Investigation into Cancer and Nutrition (EPIC) study. Public Health Nutr 2003;6:407–13. https://doi.org/10.1079/PHN2002439.

[2] InterAct Consortium, Peters T, Brage S, Westgate K, Franks PW, Gradmark A, et al. Validity of a short questionnaire to assess physical activity in 10 European countries. Eur J Epidemiol 2012;27:15–25. https://doi.org/10.1007/s10654-011-9625-y.

[3] Johansson I, Hallmans G, Wikman A, Biessy C, Riboli E, Kaaks R. Validation and calibration of food-frequency questionnaire measurements in the Northern Sweden Health and Disease cohort. Public Health Nutr 2002;5:487–96. https://doi.org/10.1079/PHNPHN2001315.

[4] Bodén S, Wennberg M, Van Guelpen B, Johansson I, Lindahl B, Andersson J, et al. Dietary inflammatory index and risk of first myocardial infarction; a prospective population-based study. Nutr J 2017;16:1–10. https://doi.org/10.1186/s12937-017-0243-8.

[5] Winkvist A, Klingberg S, Nilsson LM, Wennberg M, Renström F, Hallmans G, et al. Longitudinal 10-year changes in dietary intake and associations with cardio-metabolic risk factors in the Northern Sweden Health and Disease Study. Nutr J 2017;16:1–12. https://doi.org/10.1186/s12937-017-0241-x.
