# Supplementary material for: Impact of achievement and change in achievement of lifestyle recommendations in middle-age on risk of the most common potentially preventable cancers
Source: Prev Med. 2021 Dec;153:106712. doi: 10.1016/j.ypmed.2021.106712 (PMC8633845; doi:10.1016/j.ypmed.2021.106712)
Supplement: Appendix C — Appendix Figure C.1a Distribution of mean UK lifestyle score in the preceding 10 years and association between mean UK lifestyle score and cancer incidence in men. Appendix Figure C.1b Distribution of mean UK lifestyle score in the preceding 10 years and association between mean UK lifestyle score and cancer incidence in women. Appendix Figure C.2a Distribution of change in UK lifestyle score in the preceding 10 years (baseline to 10-year health check) and association between change in UK lifestyle score and cancer incidence in men. Appendix Figure C.2b Distribution of change in UK lifestyle score in the preceding 10 years (baseline to 10-year health check) and association between change in UK lifestyle score and cancer incidence in women. Appendix Figure C.3. Distribution of mean Nordic lifestyle score in the preceding 10 years (bars, left axis) and association between mean Nordic lifestyle score and cancer incidence (forest plot, right axis) in women, excluding breast cancer.. Appendix Figure C.4. Distribution of change in Nordic lifestyle score in the preceding 10 years (baseline to 10-year health check) (bars, left axis) and association between change in Nordic lifestyle score and cancer incidence (forest plot, right axis) in women, excluding breast cancer.. [file mmc3.pdf]

Appendix C

Figure C.1

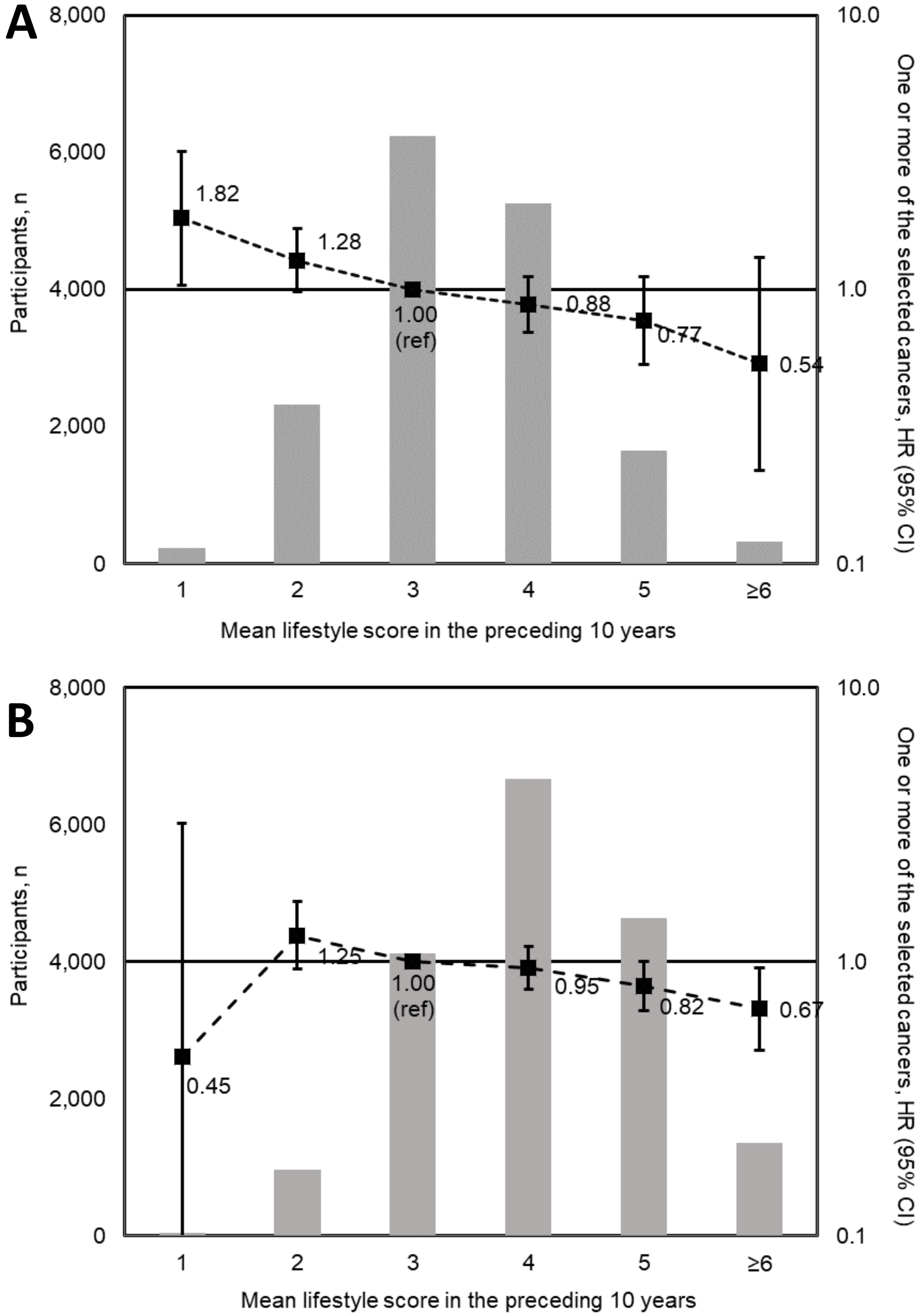

Figure C.1. Distribution of mean UK lifestyle score in the preceding 10 years (bars, left axis) and association between mean UK lifestyle score and cancer incidence (forest plot, right axis) in men (A) and women (B). HRs adjusted for age group at baseline, baseline year, education level and marital status. CI confidence interval, HR hazard ratio

Figure C.2

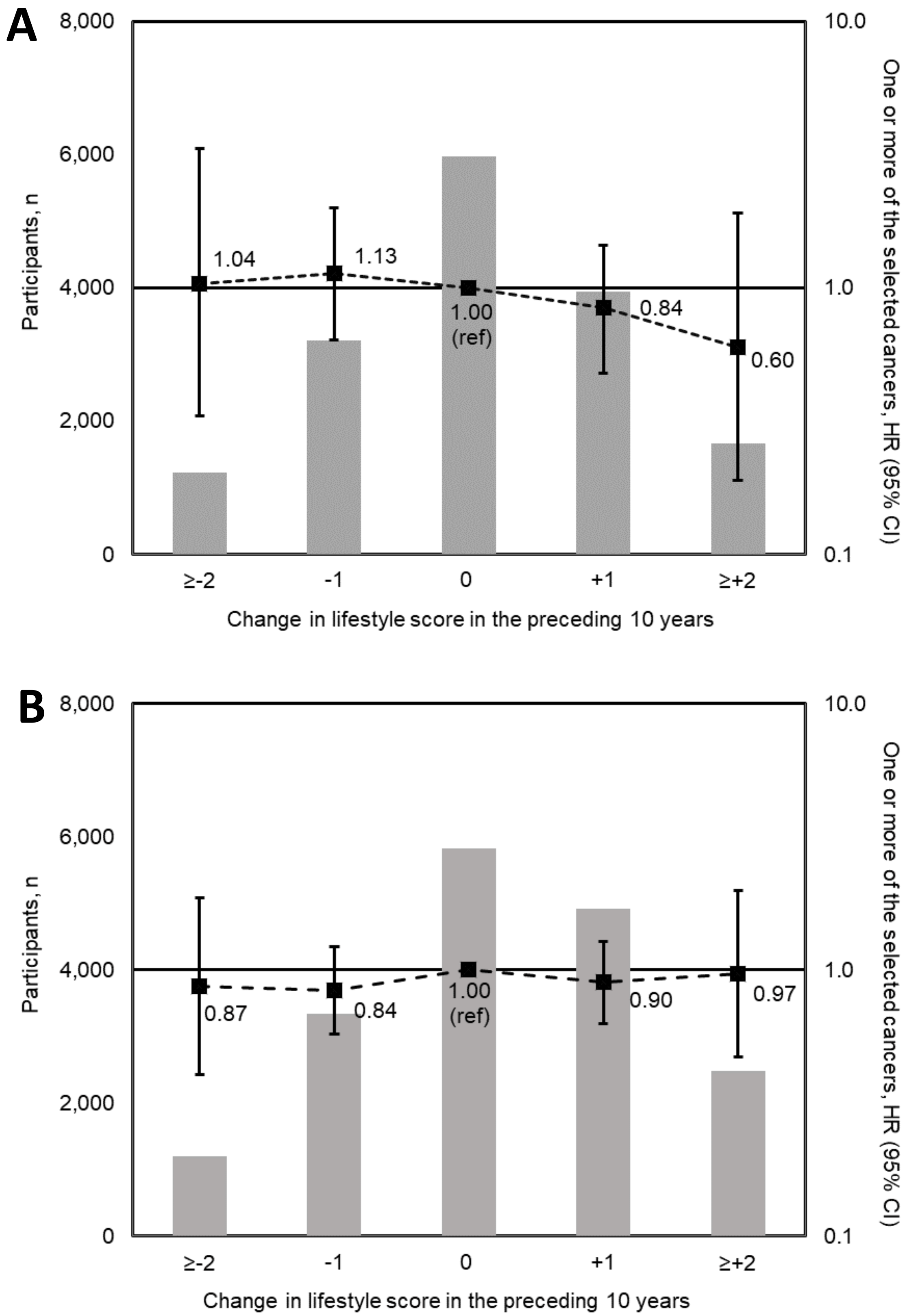

Figure C.2. Distribution of change in UK lifestyle score in the preceding 10 years (baseline to 10-year health check) (bars, left axis) and association between change in UK lifestyle score and cancer incidence (forest plot, right axis) in men (A) and women (B). HRs adjusted for age group at baseline, baseline year, education level and marital status. CI confidence interval, HR hazard ratio

Figure C.3

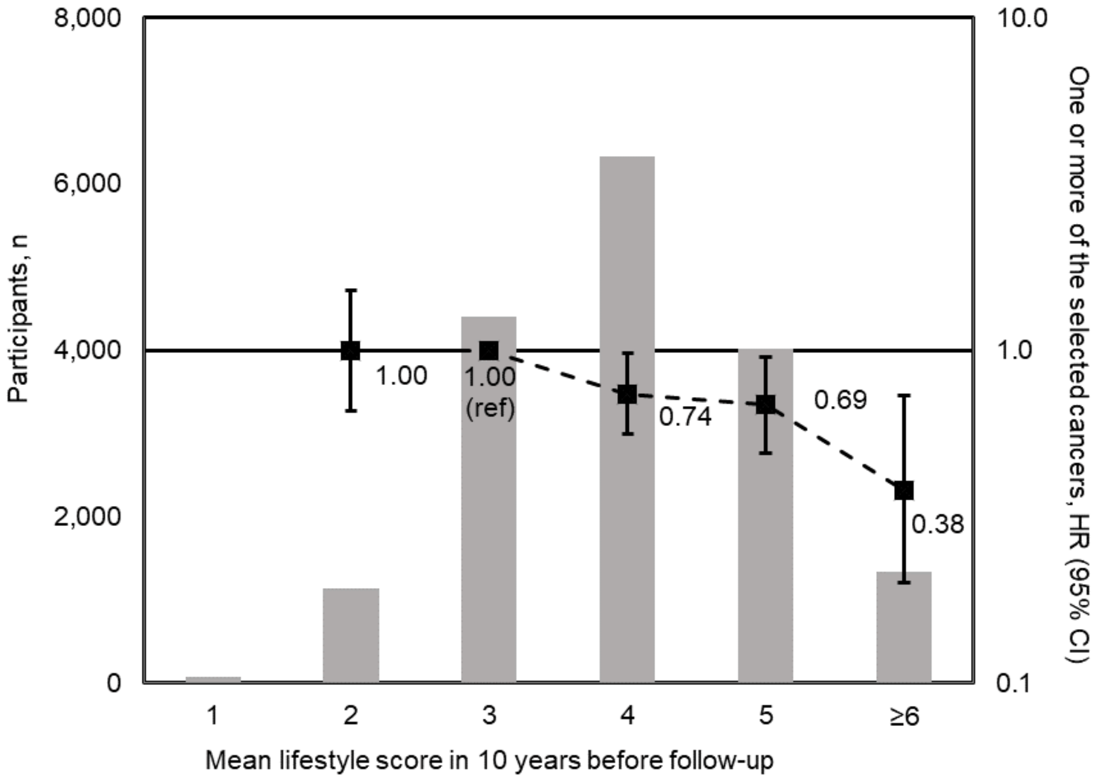

Figure C.3. Distribution of mean Nordic lifestyle score in the preceding 10 years (bars, left axis) and association between mean Nordic lifestyle score and cancer incidence (forest plot, right axis) in women, excluding breast cancer. HRs adjusted for age group at baseline, baseline year, education level and marital status. CI confidence interval, HR hazard ratio

Figure C.4

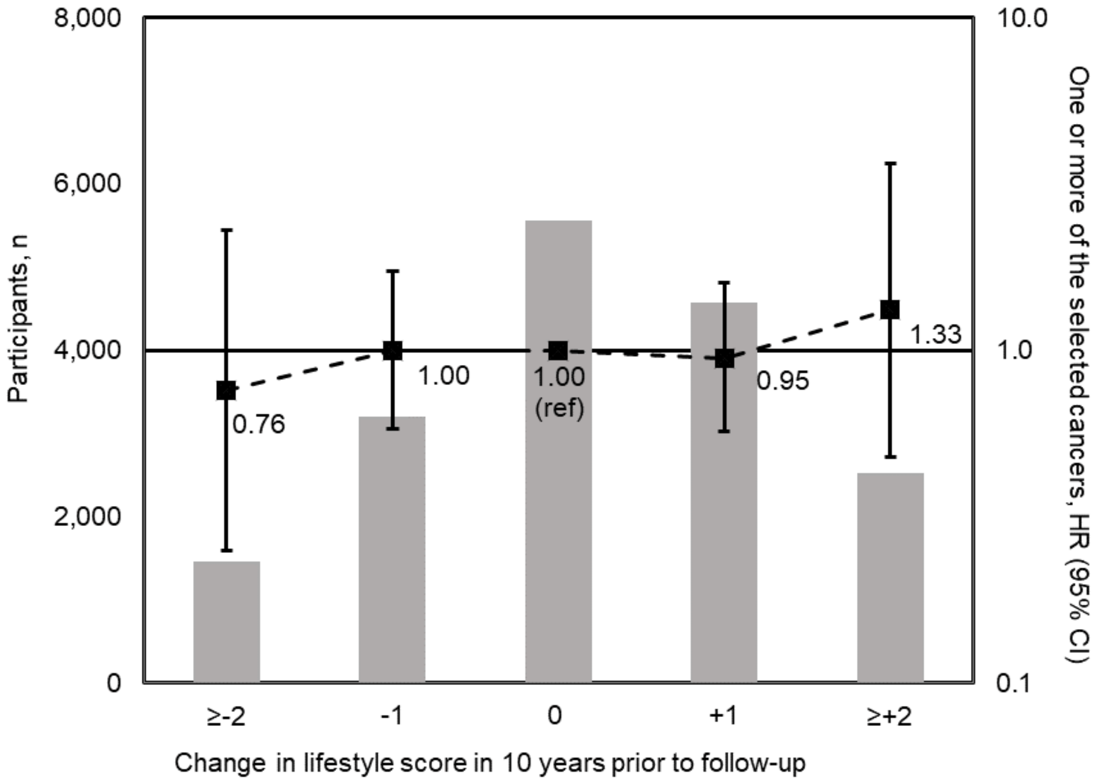

Figure C.4. Distribution of change in Nordic lifestyle score in the preceding 10 years (baseline to 10-year health check) (bars, left axis) and association between change in Nordic lifestyle score and cancer incidence (forest plot, right axis) in women, excluding breast cancer. HRs adjusted for age group at baseline, baseline year, education level and marital status. CI confidence interval, HR hazard ratio
